# Prevalence of dyslipidemia and associated factors among the hypertensive population from rural Northeast China

**DOI:** 10.1186/s12889-015-2486-7

**Published:** 2015-11-21

**Authors:** Shasha Yu, Hongmei Yang, Xiaofan Guo, Xingang Zhang, Liqiang Zheng, Yingxian Sun

**Affiliations:** Department of Cardiology, The First Hospital of China Medical University, 155 Nanjing North Street, Heping District, Shenyang, 110001 Liaoning China; Department of Clinical Epidemiology, Shenjing Hospital of China Medical University, Shenyang, Liaoning China

**Keywords:** Dyslipidemia, Hypertension, Rural, Update

## Abstract

**Background:**

Our latest study reported the grim status of hypertension in rural China with the prevalence of hypertension reached 51.1 %. However, we lack the latest data about the prevalence and epidemiological features of dyslipidemia among hypertensive residents in rural China.

**Methods:**

A cross-sectional survey was conducted from July 2012 to August 2013 through a cluster multistage sampling to a resident group of 4048 individuals (2152 men, 2896 women) with hypertension, age ≥ 35 years, in the rural Northeast China. Serum lipids level were proposed by National Cholesterol Education Program Adult Treatment Panel III.

**Results:**

Of the hypertension residents without antihypertension treatment, 34.5 % had borderline high total cholesterol, 19.2 % had high total cholesterol, 11.4 % had low high-density lipoprotein cholesterol and 37.4 % had high non HDL-C. The population with borderline high, high, and very high low-density lipoprotein cholesterol was 20.9, 6.7 and 2.3 %, respectively. In addition, 14.3 % had borderline high triglycerides, 17.4 % had high TG and 2.4 % had very high TG. The awareness rate of dyslipidemia among the study population was 5.9 %. After adjusting for independent variables, fasting plasma glucose, body mass index, Han nationality, current drinking and smoking, higher annual income and classification of blood pressure were risk factors for dyslipidemia while moderate physical activity was protective factor for dyslipidemia. On the contrary, gender and current drinking decrease the risk of HDL-C.

**Conclusion:**

The prevalence of dyslipidemia was dramatically high and dyslipidemia screening was in-need in all diagnosed hypertensive individuals.

## Background

With development of economy and improvement of living standards, more and more people surfer from atherosclerotic cardiovascular diseases (ASCVD) which constitute the leading cause of death [1]. Further more, with the progression of urbanization and industrialization, the morbidity and mortality of ASCVD which include coronary heart disease (CHD), stroke, and other atherosclerotic vascular disease increase dramatically [2]. Previous studies demonstrated that low-density lipoprotein cholesterol(LDL-C) seemingly crucial for ASCVD while high-density lipoprotein cholesterol (HDL) protect against ASCVD [3–5]. In the National lipid association recommendations for patient-centered management of dyslipidemia, they concluded that an elevated level of cholesterol, including non-HDL-C and LDL-C, is a root cause of atherosclerosis, the key underling process contributing to most of clinical ASCVD events [6].

The prevalence and epidemiologic characters of hypertension in China had been thoroughly studied in resent years and reached the conclusion that hypertension became prevalent not only in urban but also in rural areas [7]. There was high prevalence of hypertension in rural northern, northeastern, and northwestern China; but low level of awareness, and treatment rates. Blood pressure control rates were very low in all the populations studied [8–10]. Furthermore, ten year ago, hypertension is relatively more prevalent in rural Northeast China than other part of China. Our previous study revealed that the prevalence of hypertension in the rural Northeast China was 36.2 %, with 27.0, 19.8 and 0.9 % as the awareness, treatment and control rates [11]. A relatively higher rate of hypertension and paradoxically lower rates of awareness, treatment and control meant higher possibility occurrence of complications, morbidity and mortality. Most recently, we update the prevalence of hypertension in rural Northeast China, 51.1 % of residents had hypertension, with the rate of awareness, treatment and control were 43.5, 31.2 and 6 %. The prevalence of hypertension was increased acutely in the recent decade. However, there was no study focused on the temporal trend of the prevalence and updating the possible risk factors of dyslipidemia among hypertensive populations. We hypothesized that huge changes in the prevalence, risk factors of dyslipidemia among hypertensive populations might happen in rural Northeast China during this 10 years. Therefore, the objective of this study was to update the prevalence of dyslipidemia as well as their risk factors in hypertensive rural Chinese during 2012–2013.

## Methods

### Study population

Liaoning Province is located in Northeast China. Our previous study, used a cluster multistage sampling method, was also conducted in Liaoning province from October 2004 to June 2006 [11]. From January 2012 to August 2013, a representative sample of participants aged ≥ 35 years was selected to characterize the prevalence, incidence and natural history of cardiovascular risk factors in rural areas of Liaoning Province. Participants who were pregnant or had malignant tumors or mental disorders were excluded from the study. All the eligible permanent residents aged ≥ 35 years, living in their villages at least 6 months, from each village were invited to attend the study (a total of 14,016 participants). Of those, 11,956 participants agreed and completed the study to give a response rate of 85.3 %. The study was approved by the Ethics Committee of China Medical University (Shenyang, China). All procedures were performed in accordance with ethical standards. Written consent was obtained from all participants after they had been informed of the objectives, benefits, medical items and confidentiality agreement regarding their personal information. For participants who were illiterate, we obtained written informed consent from their proxies. In this report, we used only the data from participants who completed the study, which provided a final sample size of 5919 [4048 without antihypertension treatment (2152 men, 1896 women) and 1871 with antihypertension treatment (740 men and 1131women)].

### Data collection and measurements

Data were collected during a single visit to the clinic by cardiologists and trained nurses using a standard questionnaire in a face-to-face interview. Before the survey was performed, we invited all eligible investigators to attend an organized training session. The training included the purpose of this study, how to administer the questionnaire, the standard method of measurement, the importance of standardization and the study procedures. A strict test was administered after this training, and only those who scored perfectly on the test were accepted as investigators in this study. During data collection, our inspectors had further instructions and support.

Data regarding the demographic characteristics, lifestyle risk factors, dietary habits, family income and family history of chronic diseases were obtained during the interview using the standardized questionnaire. The study was guided by a central steering committee with a subcommittee for quality control. Educational level was assessed as completion of primary school or less, middle school or high school and higher. Self-reported sleep duration (including nocturnal and nap duration) was obtained from the questionnaire. The responses were categorized into four groups: ≤7, 7–8, 8–9 and >9 h/d. Family income was classified as ≤5000, 5000–20,000 and >20,000 CNY/year.

According to American Heart Association protocol, blood pressure (BP) was measured three times at 2-min intervals after at least 5 min of rest using a standardized automatic electronic sphygmomanometer (HEM-907; Omron), which had been validated according to the British Hypertension Society protocol [12]. The participants were advised to avoid caffeinated beverages and exercise for at least 30 min before the measurement. During the measurement, the participants were seated with their arms supported at the level of the heart. The mean of three BP measurements was calculated and used in all analyses.

Weight and height were measured to the nearest 0.1 kg and 0.1 cm, respectively, with the participants wearing light-weight clothing and without shoes. Waist circumference (WC) was measured at the umbilicus using a non-elastic tape (to the nearest 0.1 cm), with the participants standing at the end of normal expiration. Body mass index (BMI) was calculated as the weight in kilograms divided by the square root of the height in meters.

Fasting blood samples were collected in the morning after at least 12 h of fasting. Blood samples were obtained from an antecubital vein into Vacutainer tubes containing ethylenediaminetetraacetic acid (EDTA). Fasting plasma glucose (FPG), total cholesterol (TC), LDL-C, HDL-C, TG and other routine blood biochemical indexes were analyzed enzymatically using an autoanalyzer. All laboratory equipment was calibrated, and blinded duplicate samples were used for these analyses.

### Definitions

Hypertension was considered present if any of the following conditions were met: systolic blood pressure ≥ 140 mmHg, diastolic blood pressure ≥ 90 mmHg, or reported use of a medication for hypertension and the classification of BP in response to JNC8. Stage 1 hypertension Clinic blood pressure is 140/90 mmHg or higher. Stage 2 hypertension Clinic blood pressure is 160/100 mmHg or higher. Stage 3 hypertension hypertension Clinic blood pressure is 180 mmHg or higher [13]. TC, LDL-C. HDL-C and TG levels were classified on the basis of the National Cholesterol Education Program Adult Treatment Panel III [14]. Borderline high and high TC was defined as having TC levels of 5.17–6.20 mmol/L(200–239 mg/dL) and ≥6.21 mmol/L (≥240 mg/dL), respectively. Low HDL-C was defined as having HDL-C levels <1.03 mmol/L (<40 mg/dL). Borderline high, high and very high LDL-C was defined as having LDL-C levels of 3.38–4.15 mmol/L(130–159 mg/dL), 4.16–4.93 mmol/L(160–189 mg/dL), and ≥ 4.94 mmol/L (≥190 mg/dL), respectively. Borderline high, high and very high TG was defined as having triglyceride levels of 1.69–2.25 mmol/L (150–199 mg/dL), 2.26–5.64 mmol/L (200–499 mg/dL), and ≥5.65 mmol/L (≥500 mg/dL), respectively. Mixed hyperlipidemia was defined as TC ≥ 200 mg/dL plus TG ≥200 mg/dL. Severe hyperlipidemia was defined as TC ≥ 300 mg/dL and/or TG ≥ 500 mg/dL. Normotriglyceridemic hypoalphalipoproteinemia was defined as HDL-C <40 mg/dL and TG <200 mg/dL. According to the American Diabetes Association (ADA) criteria of 1997, impaired fasting blood glucose (IFG) was defined as FPG ≥6.1 mmol/L(<109.8 mg/dL) and <7.0 mmol/L (<126 mg/dL). According to the WHO criteria, the subjects with BMI ≥ 25 kg/m^2^ and <30 kg/m^2^ were diagnosed as overweight, and those with ≥30 kg/m^2^ as obese.

Physical activity included occupational and leisure-time physical activity. A detailed description of the methods for assessing physical activity has been presented elsewhere [15]. Occupational and leisure-time physical activity were merged and regrouped into the following three categories: 1) low—subjects who reported light levels of both occupational and leisure-time physical activity, 2) moderate—subjects who reported moderate or high levels of either occupational or leisure-time physical activity and 3) high—subjects who reported a moderate or high level of both occupational and leisure-time physical activity.

### Statistical analysis

Descriptive statistics were calculated for all the variables, including continuous variables (reported as mean values and standard deviations) and categorical variables (reported as numbers and percentages). Differences between different dyslipidemia groups were evaluated using Student’s *t*-test, ANOVA, non-parametric test or the *χ*2-test as appropriate. Multivariate logistic regression analyses were used to identify independent factors of dyslipidemia among hypertensive adults with odds ratios (ORs) and corresponding 95 % confidence intervals (CIs) calculated. All the statistical analyses were performed using SPSS version 17.0 software, and P values less than 0.05 were considered to be statistically significant [7].

## Results

### Basic characteristic of the study population

Characteristics of the hypertensive residents enrolled in this study, as stratified by gender, are shown in Table 1. All subjects selected were more than 35 years old and the average ages of the men and women were 56.40 ± 10.65 and 56.40 ± 10.65 years, respectively. For men, about 5.7 % of the residents were minority nationality, and for women, 6.1 %. Compared with men, hypertensive women had higher levels of TC, LDL-C and non HDL-C(*P* < 0.001), but current smoking and drinking, SBP, DBP and WC were significantly higher in men than in women (*P* < 0.001).

**Table 1:** Characteristics of study population by gender in the rural hypertensive population of Liaoning Province, China

	Without antihypertension treatment			With antihypertension treatment		
Variables	Total (4048)	Men (2152)	Women (1896)	Total (1871)	Men (740)	Women (1131)
Age (year)	56.44 ± 10.37	56.40 ± 10.65	56.48 ± 10.04	59.14 ± 9.73	59.65 ± 10.20	58.81 ± 9.40
Systolic Blood Pressure (mmHg)	157.63 ± 17.00#	158.18 ± 17.42	157.01 ± 16.50	161.51 ± 24.22	161.43 ± 22.92	161.43 ± 25.05
Diastolic Blood Pressure (mmHg)	88.31 ± 10.51*	89.75 ± 10.60	86.68 ± 10.17	89.95 ± 12.40*	91.75 ± 12.55	88.77 ± 12.17
Body mass index (kg/m^2^)	25.19 ± 3.61	25.12 ± 3.52	25.26 ± 3.71	26.37 ± 3.64#	26.08 ± 3.46	26.57 ± 3.74
WC (cm)	84.00 ± 9.74*	85.15 ± 9.58	82.71 ± 9.77	86.80 ± 9.44*	88.01 ± 9.66	86.02 ± 9.22
Total Cholesterol (mmol/L)	5.37 ± 1.07*	5.31 ± 1.04	5.43 ± 1.09	5.53 ± 1.20*	5.31 ± 1.09	5.67 ± 1.25
Triglycerides (mmol/L)	1.75 ± 1.69	1.76 ± 1.75	1.73 ± 1.53	2.02 ± 1.57#	1.90 ± 1.58	2.09 ± 1.56
LDL-C (mmol/L)	3.03 ± 0.82*	2.96 ± 0.81	3.10 ± 0.84	3.19 ± 0.92*	3.07 ± 0.86	3.26 ± 0.95
HDL-C (mmol/L)	1.46 ± 0.42	1.47 ± 0.48	1.45 ± 0.36	1.33 ± 0.35*	1.30 ± 0.36	1.36 ± 0.35
Non-HDL (mg/dl)	150.48 ± 41.02*	147.77 ± 40.27	153.53 ± 41.66	161.68 ± 44.39*	154.82 ± 40.95	166.17 ± 45.98
Fasting Plasma Glucose (mmol/L)	6.08 ± 1.85	6.11 ± 1.87	6.04 ± 1.83	6.34 ± 1.95	6.23 ± 1.81	6.41 ± 2.04
Ethnicity^a^	3809 (94.1)	2029 (94.3)	1780 (93.9)	1795 (95.9)	713 (96.4)	1082 (95.7)
Current smoking status (Yes)	1552 (38.3)*	1208 (56.1)	344 (18.1)	524 (72.0)*	343 (46.4)	181 (16.0)
Current drinking status (Yes)	1205 (29.8)*	1127 (52.4)	78 (4.1)	245 (13.1)*	225 (30.4)	20 (1.8)

Data are expressed as the mean ± SD or as n (%). *Abbreviations*: *LDL-C* low-density lipoprotein cholesterol, *HDL-C* high-density lipoprotein cholesterol; ^a^Including some ethnic minorities in China, such as Mongol and Manchu

^#^means *P*<0.05

^*^means *P*<0.001

### Prevalence of dyslipidemia

Table 2 shows the prevalence of serum cholesterol and triglyceride by age an gender group. Prevalence of borderline high and high TC was 34.5 and 19.2 %, respectively, and the prevalence for both was significantly higher in women than in men (*P* < 0.001). The age-specific prevalence of borderline high TC for women increased with increased age (*P* < 0.001) while high TC increased with increased age for both genders (*P* < 0.001). The prevalence of low HDL-C was 11.4 %. The prevalence of low HDL-C was significantly higher in men than in women (13.9 % vs. 8.5 %, *P* < 0.001). The prevalence of borderline high, high and very high LDL-C was 20.9, 6.7, and 2.3 %, respectively. The prevalence of borderline high, high and very high LDL-C was slightly higher in women than in men (*P* < 0.05). In addition, the prevalence of borderline high, high and very high TG was 14.3, 17.4 and 2.4 %, respectively. Among the high and very high group, the age-specific prevalence of men decrease from 35 years old (*P* < 0.001); however, it increased in the group of women in the borderline high and high group (*P* < 0.001). Women had higher prevalence of non HDL-C than men(40.4 % vs. 30.7 %). The age-specific prevalence gradually decrease from 35 years old in the group of men while on the contrary significantly increase in women. The prevalence of serum cholesterol and triglycerides by cardiovascular co-morbidities are presented in Table 3. The prevalence of dyslipidemia was increased with the severity of co-morbidities in the borderline, high and very high group. It was significant in the HDL-C, non HDL-C, LDL-C and TG only.

**Table 2:** Prevalence of dyslipidemia by gender and age group in the hypertensive rural population without antihypertension treatment

	TC mmol/l(mg/dl)		HDL-C mmol/L(mg/dl)	LDL-C mmol/L(mg/dl)			TG mmol/L(mg/dl)			Non-HDL (mg/dl)
	5.17–6.2 (200–239)	≥6.21 (≥240)	<1.03 (<40)	3.8–4.15 (130–159)	4.16–4.93 (160–189)	≥4.49 (≥190)	1.69–2.25 (150–199)	2.26–5.64 (200–499)	≥5.65 (≥500)	≥160
Men										
Age (years)										
≥35	716 (33.3)	385 (17.9)	299 (13.9)	402 (18.7)	117 (5.4)	49 (2.3)	274 (12.7)	383 (17.8)	60 (2.8)	746 (34.7)
35–45	114 (32.4)	62 (17.6)	53 (15.1)	71 (20.2)	20 (17.1)	5 (10.2)	43 (12.2)	98 (27.8)	15 (4.3)	133 (37.8)
45–55	200 (33.4)	127 (21.2)	89 (14.9)	128 (21.4)	38 (6.3)	12 (2.0)	76 (12.7)	131 (21.9)	22 (3.7)	231 (38.6)
55–65	250 (32.9)	140 (18.4)	102 (13.4)	132 (17.4)	41(5.4)	24 (3.2)	102(13.4)	111 (14.6)	20 (2.6)	260(34.2)
≥65	152 (34.4)	56 (12.7)	55 (12.4)	71 (16.1)	18 (4.1)	8 (1.8)	53 (12.0)	43 (9.7)	3 (0.7)	122(27.6)
P for trend	0.786	0.003	0.621	0.076	0.313	0.241	0.376	<0.001	<0.001	0.002
Women										
Age (years)										
≥35	682 (36.0)	394 (20.8)	162 (8.5)	443 (23.4)	155 (8.2)	45 (2.4)	304 (16.0)	321 (16.9)	37 (2.0)	766 (40.4)
35–45	63 (23.3)	19 (7.0)	27 (10.0)	34 (12.5)	7 (2.6)	3 (1.1)	30 (11.1)	32 (11.8)	3 (1.1)	49 (18.1)
45–55	208 (36.4)	88 (15.4)	53 (9.3)	124 (21.7)	28 (4.9)	13 (2.3)	97 (17.0)	78 (13.7)	13 (2.3)	197 (34.5)
55–65	250 (36.7)	195 (28.6)	54 (7.9)	192 (28.2)	80 (11.7)	17 (2.5)	115 (16.9)	136 (20.0)	14 (2.1)	339 (49.8)
≥65	161 (43.2)	92 (24.7)	28 (7.5)	93 (24.9)	40 (10.7)	12 (3.2)	62 (16.6)	75 (20.1)	7 (1.9)	181 (48.5)
P for trend	<0.001	<0.001	0.585	<0.001	<0.001	0.130	0.028	<0.001	0.575	<0.001

Data are n(%). *TC*total cholesterol, *HDL-C* high-density lipoprotein, *LDL-C* low-density lipoprotein, *TG* triglyceride

**Table 3:** Prevalence of dyslipidemia by cardiovascular co-morbidities in the rural population without antihypertension treatment

	TC mmol/l(mg/dl)		HDL-C mmol/L(mg/dl)	LDL-C mmol/L(mg/dl)			TG mmol/L(mg/dl)			Non-HDL (mg/dl)
Cardiovascular co-morbidities	5.17–6.2 (200–239)	≥6.21 (≥240)	<1.03 (<40)	3.8–4.15 (130–159)	4.16–4.93 (160–189)	≥4.49 (≥190)	1.69–2.25 (150–199)	2.26–5.64 (200–499)	≥5.65 (≥500)	≥160
Men										
IFG	155 (38.5)	83 (20.6)	56 (13.9)#	90 (22.3)	28 (6.9)	9 (2.2)	61 (15.1)	75 (18.6)*	7 (1.7)*	167 (41.4)*
DM	92 (35.5)	62 (23.9)	49 (18.9)	59 (22.8)	15 (5.8)	10 (3.9)	28 (10.8)	87 (33.6)	23 (8.9)	130 (50.2)
Overweight	296 (33.7)	184 (20.9)	163 (18.5)*	183 (20.8)#	74 (8.4)	26 (3.0)	137 (15.6)	213 (24.2)*	34 (3.9)	373 (42.4)*
Obesity	68 (40.2)	30 (17.8)	47 (27.8)	51 (30.2)	10 (5.9)	3 (1.8)	22 (13.0)	65 (38.5)	7 (4.1)	82 (48.5)
Women										
IFG	99 (35.1)	76 (27.0)	35 (12.4)#	72 (25.5)	36 (12.8)	1 (0.4)#	53 (18.8)	47 (16.7)*	8 (2.8)	142 (50.4)*
DM	77 (34.1)	75 (33.2)	21 (9.3)	70 (31.0)	26 (11.5)	7 (3.1)	32 (14.2)	68 (30.1)	10 (4.4)	124 (54.9)
Overweight	269 (35.4)	168 (22.1)	72(9.5)#	202(26.6)	65(8.6)	22(2.9)	131(17.2)	180(23.7)	15(2.0)	336(44.2)#
Obesity	60 (32.1)	43 (23.0)	24(12.8)	39(20.9)	23(12.8)	7(3.7)	35(18.7)	49(26.2)	3(1.6)	81(43.3)

*IFG* impaired fasting blood glucose, *DM* diabetes mellitus. Data are n(%). *TC* total cholesterol, *HDL-C*high-density lipoprotein, *LDL-C* low-density lipoprotein, *TG* triglyceride

*IPG compared with DM (*P* < 0.05), # overweight compared with obesity (*P* < 0.05)

**Fig. 1:**
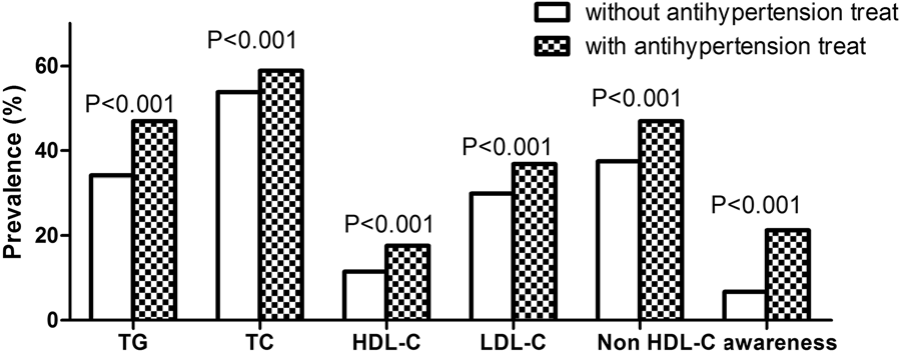
Prevalence of different dyslipidemia in hypertensive residents with or without antihypertension treatment. TC, total cholesterol; HDL-C, high-density lipoprotein; LDL-C, low-density lipoprotein; TG, triglyceride

Figure 1 Show that residents with antihypertension treatment have significantly higher prevalence of dyslipidemia than those without treatment (TG: 34.1 % vs. 46.9 %, TC: 53.8 % vs. 58.9 %, HDL-C: 11.4 % vs. 17.5 %, LDL-C: 29.9 % vs. 36.8 %, Non HDL-C: 37.4 % vs. 46.9 %, all *P* < 0.001). Besides, the awareness and control rate among those with antihypertension treatment is statically higher than those without it (19.0 % vs. 5.9 %, *P* < 0.001, 10.7 % vs. 2.5 %, *P* <0.001).

**Fig. 2:**
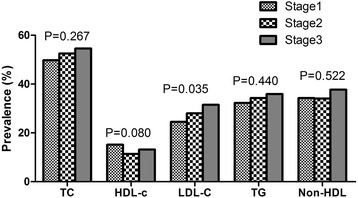
Prevalence of serum cholesterol and triglyceride by classification of blood pressure in men. TC, total cholesterol; HDL-C, high-density lipoprotein; LDL-C, low-density lipoprotein; TG, triglyceride

**Fig. 3:**
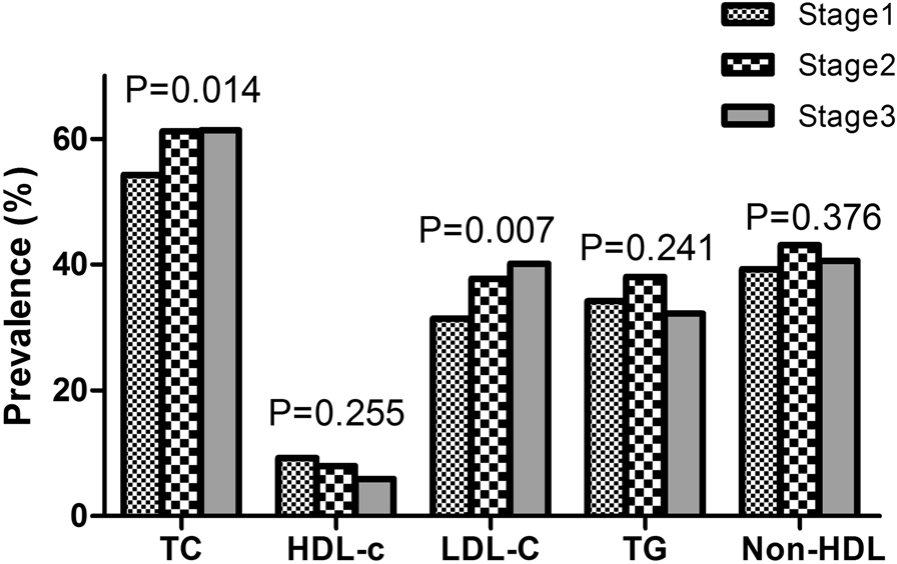
Prevalence of serum cholesterol and triglyceride by classification of blood pressure in women. TC, total cholesterol; HDL-C, high-density lipoprotein; LDL-C, low-density lipoprotein; TG, triglyceride

The prevalence of LDL-C increased with the increased stage of BP and showed a significant difference among the three stages in both men and women. In contrast, the prevalence of HDL-C decreased with the increased stage of BP but did not reach significant difference in women (*P* = 0.255). Besides, we also see a trend between HDL-C and the stage of BP in men and TC and the stage of BP in women (Fig. 2). The prevalence of TC and LDL-C increased with the increased stage of BP and showed a significant difference among the three stages. However, we did not see a trend between HDL-C and the stage of BP, and TG and the stage of BP. Similarly, there was no trend existed between Non HDL-C and the stage of BP (Fig. 3).

### Associated factors of serum cholesterol and triglycerides

Table 4 presents the result of multiple logistic analyses of serum total, LDL, HDL, non HDL-C cholesterol and TG, respectively. After adjusting for independent variables, fasting plasma glucose(FPG), body mass index(BMI), Han nationality, current drinking and smoking, higher annual income and classification of blood pressure were risk factor for dyslipidemia while moderate physical activity was protective factor for dyslipidemia. On the contrary, gender and current drinking decrease the risk of HDL-C.

**Table 4:** Multiple logistic regression analysis of serum cholesterol, triglycerides, and the associated factors in the rural population without antihypertension treatment

Variable	OR	95 % CI	*P* values
TC			
Gender^a^	1.693	1.445,1.985	<0.001
Age (years)	1.022	1.015,1.030	<0.001
Han nationality^b^	1.529	1.164,2.008	<0.001
Current drinking	1.898	1.600,2.252	<0.001
Fasting plasma glucose(mmol/L)	1.107	1.063,1.153	<0.001
BMI (kg/m^2^)	1.044	1.025,1.064	<0.001
Annual income >20000 CNY/year^c^	1.381	1.115,1.710	0.003
HDL-C			
Gender^a^	0.336	0.264,0.427	<0.001
Current smoking	1.321	1.054,1.656	0.016
Current drinking	0.246	0.186,0.327	<0.001
Fasting plasma glucose(mmol/L)	1.049	1.010,1.089	0.014
BMI (kg/m^2^)	1.135	1.104,1.167	<0.001
Moderate physical activity^e^	0.740	0.587,0.932	0.011
LDL-C			
Gender^a^	1.650	1.385,1.966	<0.001
Age(years)	1.019	1.011,1.028	<0.001
Current drinking	1.217	1.008,1.469	0.041
Fasting plasma glucose(mmol/L)	1.084	1.046,1.123	<0.001
BMI (kg/m^2^)	1.085	1.046,1.107	<0.001
≥ Middle school educational status	1.311	1.015,1.694	0.038
Stage 3 of BP classification^d^	1.254	1.009,1.559	0.041
Moderate physical activity^e^	0.835	0.712,0.980	0.028
Annual income >20000 CNY/year^c^	1.318	1.042,1.668	0.021
TG			
Current smoking	1.200	1.025,1.404	0.023
Fasting plasma glucose(mmol/L)	1.160	1.116,1.207	<0.001
BMI (kg/m^2^)	1.155	1.132,1.179	<0.001
Moderate physical activity^e^	0.821	0.700,0.963	0.015
Non-HDL			
Gender^a^	1.308	1.110,1.542	0.001
Age(years)	1.019	1.011,1.026	<0.001
Han nationality^b^	1.724	1.268,2.343	0.001
Fasting plasma glucose(mmol/L)	1.147	1.103,1.192	<0.001
BMI (kg/m^2^)	1.097	1.076,1.119	<0.001
Moderate physical activity^e^	0.856	0.734,0.998	0.048
Annual income 5000–20000 CNY/year^c^	1.260	1.031,1.541	0.024
Annual income >20000 CNY/year^c^	1.569	1.254,1.963	<0.001

*OR* odds ratio, *CI* confidence interval, *TC* total cholesterol, *HDL-C* high-density lipoprotein, *LDL-C* low-density lipoprotein, *TG* triglyceride, *BMI* body mass index

Adjusted for gender, age, ethnic, education, current smoking, current drinking, fasting plasma glucose, BMI, BP classification, sleep duration, physical activity and annual income and the variables presented in this table *P* values <0.05, but the variables with *P* values >0.05 were not shown in this table

^a^contrast to men

^b^contrast to some ethnic minorities in China, such as Mongol and Manchu

^C^contrast to Annual income ≤5000 CNY/year

^d^contrast to Stage 1 of BP classification

^e^ contrast to light physical activity

## Discussion

The main finding of this study was that during the past ten years, in rural areas of China, the prevalence of dyslipidemia in hypertensive was still high, and some kind of it even worse. In the present study, we found that the prevalence of isolated hypercholesterolemia (TC ≥ 240 mg/dL and TG <200 mg/dL), low HDL-C concentration, increased LDL-C concentration (LDL-C ≥ 160 mg/dL), isolated hypertriglyceridemia (TG ≥ 200 mg/dL) was 12.5, 11.4, 9.0 and 19.8 %, respectively. The prevalence of mixed hyperlipidemia (TC ≥ 200 mg/dL plus TG ≥200 mg/dL), severe hyperlipidemia (TC ≥ 300 mg/dL and/or TG ≥ 500 mg/dL), normotriglyceridemic hypoalphalipoproteinemia (HDL-C <40 mg/dL and TG <200 mg/dL) was 13.7, 4.1 and 6.2 %, respectively. Furthermore, 68.7 % of adult hypertensive had at least one type of dyslipidemia (including borderline high, high and very high TC, LDL-C, TG and low HDL-C) in rural China. Despite the high prevalence of dyslipidemia in rural Northeast China, the awareness for those without antihypertension treatment was only 5.9 %. However, for those received antihypertension treatment, the awareness rate increased to 19.0 %. Surprisingly, the prevalence of dyslipidemia among those with antihypertension treatment were significantly higher than those without antihypertension treatment (all *P* < 0.001).

Our study found that dyslipidemia was prevalent in rural hypertensive Chinese compared with those living in other areas of China. Luo JY and colleagues reported that the prevalence of dyslipidemia in the Northwestern China was 52.72 % [16]. One recent meta-analysis claimed that the prevalence, awareness, treatment, and control rates of dyslipidemia in China were 41.9 % (95 % CI:37.7–46.2 %), 24.4 % (95 % CI:14.4–38.4 %), 8.8 % (95 % CI: 7.7–10.0 %), and 4.3 % (95 % CI:4.1–4.5 %), respectively [17]. Study held in Turkey claimed that among their study population 43 % had high TC(55.4 % for ours), 41.5 % had low HDL-C(13.3 % for ours), 36.2 % had high LDL-C(32.1 % for ours), and 35.7 % had high TG (38.1 % for ours) [18]. Furthermore, the prevalence of dyslipidemia in rural Northeast China was closed to many well developed countries like Canada (45 %), Korea (37.4 %), German (64.5 % for men and 65.7 % for women) [19–21]. However, compared with the relatively higher prevalence of dyslipidemia, the low awareness rate among rural Northeast Chinese worth our attention. In our study, we reported that the overall awareness rate of dyslipidemia was 10.1 % which was far lower than the other areas of China [16, 17, 22]. Surprisingly, the awareness rate in hypertensives with antihypertension was significantly higher than those without antihypertension treatment (19.0 % vs. 5.9 %, *P* <0.001). The possible reason might be those hypertensive with antihypertension treatment paid more attention to their health. They were more willingly to complete the chronic diseases related health check and take measurement to intervene.

Interestingly, data from our study revealed that even though the awareness and treatment rates in hypertensive with antihypertension treatment are higher than those without antihypertension treatment (19.0 % vs. 5.9 %, *P* < 0.001, 10.7 % vs. 2.5 %, *P* <0.001). The mean values of TG, TC, LDL-C and HDL-C in those hypertensives with antihypertension treatment are higher. Besides, the prevalence of different kind of dyslipidemia among those with antihypertension treatment are statically higher (TG: 46.9 % vs. 34.1 %; TC: 58.9 % vs. 53.8; HDL-C:17.5 % vs. 11.4 %; LDL-C: 36.8 % vs. 29.9 %; Non HDL-C: 46.9 % vs. 37.4, all *P* < 0.001). The possible reason might be: First, in our study, those with antihypertension treatment had higher value of age, SBP, DBP and BMI than those without treatment (all *P* < 0.001). Many previous studies had proved that dyslipidemia was relevant to older age, hypertension and overweight/obesity [22, 23]. Secondly, rural residents still lived a relatively lower living standard in compared to the urban citizen. In rural areas, the most commonly used and cheapest anti-hypertensive drugs was diuretic which has an impact on lipid and glucose metabolism with a resultant of higher prevalence of dyslipidemia and fasting blood glucose abnormality. Thirdly, we found that the current smoking rates in hypertensive residents with antihypertension treatment is almost twice more than those without treatment. Studies had estimated that smoking might increase the risk of dyslipidemia in both women and men [24].

In our study, after adjusted for independent variables, we found that fasting plasma glucose and BMI was the common risk factors of dyslipidemia. As previous study had confirmed diabetes and overweight/obesity increased the risk of dyslipidemia [22, 23]. Besides, older age and women was also risk factors of dyslipidemia except for low HDL-C. It is in accordance with our previous study [25]. Consistent with previous study, current smoking and drinking increase the risk of dyslipidemia in hypertensive population. But our study reached the conclusion that current drinking might protect hypertensives from low HDL-C. Choudhury SR and colleagues investigated that alcohol drinkers had a higher HDL-cholesterol level than non-drinkers [26]. This conclusion might partially account for why current drinking is the protective factor for HDL-C. Genetic and lifestyle differences might be responsible for the higher risk of Han nationality for dyslipidemia. Higher annual income was correlated with an increased risk of TC, LDL-C and non HDL-C in our study; however, the relationship between income and dyslipidemia is still controversial. Study held in Korea claimed that household income level was positively associated with prevalence of dyslipidemia in men but negatively linked to decrease risk of dyslipidemia in women [20]. Moderate physical activity help to reduce the risk of dyslipidemia and had been recommended as lifestyle therapies for dyslipidemia [27].

Our study has several limitations. The major limitation is the cross-sectional design which reflects only associations between dyslipidemia and risk factors, but unable to observe prospectively. Besides, in this study, the sample contains only hypertensive, lacking normotensive for comparison. The prevalence of dyslipidemia was based on a single assessment of blood samples, which may introduce error. In addition, although the researchers had been trained according to a standardized protocol of measurements, measurements at a single visit might lead to incorrect values for the anthropometric indexes. And In this study, the sample contained only hypertensives, lacking normotensives for comparison. Finally, we did not evaluate the medication of antihypertension which might be crucial for the evaluation of the characters of dyslipidemia.

## Conclusion

In summary, our population-based study updated the latest prevalence of dyslipidemia in rural Northeast China and revealed a higher prevalence of dyslipidemia in rural areas. Besides, the apparently lower awareness, treatment rates need our attention. In addition to some of the uncontrollable risk factors associated with dyslipidemia, including age, gender, ethnic, we should pay attention to the risk factors that can be intervened, like recommendation of controlling of blood glucose, quite smoking and drinking. Besides, we should strengthen the dyslipidemia screening for residents with higher risk like women and Han nationality.

## Abbreviations

TC: Total cholesterol
HDL-C: High-density lipoprotein cholesterol
LDL-C: Low-density lipoprotein cholesterol
TG: Triglyceride
FPG: Fasting plasma glucose
BMI: Body mass index
ASCVD: Atherosclerotic cardiovascular diseases
CNY: China Yuan
WC: Waist circumference
SBP: Systolic blood pressure
DBP: Diastolic blood pressure
